# Supplementary material for: Kiwifruit Vine Decline Syndrome (KVDS) Alters Soil Enzyme Activity and Microbial Community
Source: Microorganisms. 2024 Nov 16;12(11):2347. doi: 10.3390/microorganisms12112347 (PMC11596200; doi:10.3390/microorganisms12112347)
Supplement: Supplementary file 1 [file microorganisms-12-02347-s001.zip › microorganisms-3275884-supplementary.pdf]

Table S1. Two-way ANOVA of the different measured soil parameters in spring.

	Orchard		Status		Orchard×Status	
	F	Sig.	F	Sig.	F	Sig.
pH	54.042	*P*<0.001	10.362	*P*<0.01	18.464	*P*<0.01
EC	0.043	·	6.032	*P*<0.05	49.154	*P*<0.001
WSC	18.670	*P*<0.001	5.199	*P*<0.05	0.253	·
WSN	1.161	·	0.073	·	135.264	*P*<0.001
NH_4^+	1.100	·	2.454	·	6.454	*P*<0.05
N total	52.427	*P*<0.001	3.279	·	2.094	·
C total	64.236	*P*<0.001	8.087	*P*<0.05	33.319	*P*<0.001
SOC	27.215	*P*<0.001	4.704	·	4.341	·
$CaCO_3$	0.235	·	0.476	·	0.466	·
C/N	3.748	·	5.233	*P*<0.05	7.527	*P*<0.05
β-Glucosidase	6.781	*P*<0.05	4.795	*P*<0.05	21.782	*P*<0.001
Phosphatase	3.741	·	26.074	*P*<0.001	8.164	*P*<0.05
Urease	13.554	*P*<0.01	2.597	·	0.067	·
BSR	7.015	*P*<0.05	81.636	*P*<0.001	24.643	*P*<0.001
Gram+	53.879	*P*<0.001	24.018	*P*<0.001	5.224	*P*<0.05
Gram-	13.181	*P*<0.01	13.312	*P*<0.01	28.172	*P*<0.001
Fungi	5.845	*P*<0.05	41.383	*P*<0.001	1.208	·
Bacteria	42.139	*P*<0.001	20.708	*P*<0.001	22.805	*P*<0.001
Actinobacteria	6.515	*P*<0.05	14.772	*P*<0.01	3.614	·
Total PLFAs	12.918	*P*<0.01	11.254	*P*<0.01	6.316	*P*<0.05
G+/G-	11.342	*P*<0.01	2.155	·	6.367	*P*<0.05
F/B	0.169	·	42.316	*P*<0.001	0.617	·

Table S2. Two-way ANOVA of the different measured soil parameters in autumn.

	Orchard		Status		Orchard×Status	
	F	Sig.	F	Sig.	F	Sig.
pH	26.949	*P*<0.001	11.452	*P*<0.01	21.057	*P*<0.001
EC	21.598	*P*<0.001	3.646	·	0.907	·
WSC	88.570	*P*<0.001	0.000	·	60.875	*P*<0.001
WSN	63.884	*P*<0.001	3.733	·	0.250	·
NH_4^+	14.981	*P*<0.01	3.247	·	2.955	·
N total	37.518	*P*<0.001	10.037	*P*<0.01	1.285	·
C total	32.671	*P*<0.001	9.708	*P*<0.01	0.309	·
SOC	33.007	*P*<0.001	11.482	*P*<0.01	0.074	·
$CaCO_3$	0.987	·	1.442	·	0.931	·
C/N	1.706	·	4.733	·	2.180	·
β-Glucosidase	10.311	*P*<0.01	6.975	*P*<0.05	0.000	·
Phosphatase	58.307	*P*<0.001	3.318	·	3.628	·
Urease	14.291	*P*<0.01	1.221	·	9.427	*P*<0.01
BSR	58.143	*P*<0.001	17.396	*P*<0.01	14.698	*P*<0.01
Gram+	17.689	*P*<0.01	2.908	·	4.892	*P*<0.05
Gram-	5.139	*P*<0.05	4.625	·	6.143	*P*<0.05
Fungi	0.476	·	0.058	·	3.593	·
Bacteria	13.887	*P*<0.01	5.115	*P*<0.05	5.653	*P*<0.05
Actinobacteria	0.014	·	0.554	·	5.639	*P*<0.05
Total PLFAs	3.519	·	0.906	·	0.539	·
G+/G-	3.563	·	0.734	·	0.099	·
F/B	4.180	·	0.001	·	0.189	·

Table S3. Three-way ANOVA of the soil parameters.

	Orchard (O)		Status (S)		Season (t)		O×S		O×t		S×t		O×S×t	
	F	Sig.	F	Sig.	F	Sig.	F	Sig.	F	Sig.	F	Sig.	F	Sig.
pH	80.382	*P*<0.001	21.557	*P*<0.001	13.559	*P*<0.01	38.986	*P*<0.001	5.127	*P*<0.05	0.075	·	0.103	·
EC	6.594	*P*<0.05	9.538	*P*<0.01	103.286	*P*<0.001	42.280	*P*<0.001	4.899	*P*<0.05	1.263	·	30.495	*P*<0.001
WSC	34.258	*P*<0.001	1.388	·	1648.290	*P*<0.001	41.409	*P*<0.001	105.965	*P*<0.001	1.358	·	48.323	*P*<0.001
WSN	53.570	*P*<0.001	2.202	·	36.186	*P*<0.001	33.982	*P*<0.001	37.957	*P*<0.001	3.149	·	44.531	*P*<0.001
NH_4^+	0.087	·	0.774	·	0.436	·	3.334	·	5.172	*P*<0.05	4.309	*P*<0.05	8.804	*P*<0.01
N total	83.130	*P*<0.001	13.250	*P*<0.01	11.063	*P*<0.01	0.019	·	1.130	·	2.643	·	3.052	·
C total	73.529	*P*<0.001	16.222	*P*<0.001	43.336	*P*<0.001	3.778	·	3.163	·	2.612	·	8.709	*P*<0.01
SOC	60.011	*P*<0.001	15.995	*P*<0.001	11.840	*P*<0.01	1.200	·	1.447	·	1.636	·	2.304	·
$CaCO_3$	1.129	·	1.833	·	10.310	*P*<0.01	0.068	·	0.172	·	0.186	·	1.378	·
C/N	5.387	*P*<0.05	9.936	*P*<0.01	18.347	*P*<0.001	9.279	*P*<0.01	0.394	·	0.110	·	1.281	·
β-Glucosidase	16.254	*P*<0.001	11.137	*P*<0.01	14.367	*P*<0.001	4.235	·	2.987	·	1.960	·	4.287	*P*<0.05
Phosphatase	60.345	*P*<0.001	13.866	*P*<0.01	66.447	*P*<0.001	0.351	·	38.566	*P*<0.001	0.152	·	8.376	*P*<0.01
Urease	27.841	*P*<0.001	3.687	·	42.923	*P*<0.001	3.972	·	0.006	·	0.126	·	5.557	*P*<0.05
BSR	29.074	*P*<0.001	64.342	*P*<0.001	88.161	*P*<0.001	0.909	·	63.278	*P*<0.001	0.520	·	33.141	*P*<0.001
Gram+	63.666	*P*<0.001	20.180	*P*<0.001	1.559	·	0.033	·	2.591	·	3.648	·	10.034	*P*<0.01
Gram-	16.713	*P*<0.001	16.093	*P*<0.001	6.947	*P*<0.05	2.530	·	0.429	·	0.570	·	28.556	*P*<0.001
Fungi	1.653	·	20.383	*P*<0.001	10.143	*P*<0.01	0.251	·	4.984	*P*<0.05	23.488	*P*<0.001	4.410	*P*<0.05
Bacteria	47.836	*P*<0.001	20.892	*P*<0.001	6.968	*P*<0.05	1.072	·	1.033	·	0.980	·	23.040	*P*<0.001
Actinobacteria	3.606	·	10.598	*P*<0.01	34.851	*P*<0.001	0.102	·	2.992	·	4.878	*P*<0.05	9.129	*P*<0.01
Total PLFAs	14.234	*P*<0.001	8.515	*P*<0.01	0.005	·	1.195	·	0.883	·	2.191	·	4.848	*P*<0.05
G+/G-	14.814	*P*<0.001	0.948	·	0.322	·	4.661	*P*<0.05	5.137	*P*<0.05	2.863	·	5.871	*P*<0.05
F/B	3.167	·	19.729	*P*<0.001	33.351	*P*<0.001	0.727	·	1.492	·	19.316	*P*<0.001	0.046	·

Table S4. Two-way ANOVA of the alpha diversity (Richness and Shannon indexes) for fungal and bacterial communities in soils in both sampling times (spring and autumn).

			Orchard (O)		Status (S)		Orchard×Status	
			F	Sig.	F	Sig.	F	Sig.
**Spring**	***Fungi***	Richness	5.316	*P*<0.05	0.079	·	1.090	·
		Shannon	7.765	*P*<0.05	1.104	·	0.629	·

	Bacteria	Richness	3.391	·	24.537	*P*<0.001	0.002	·
		Shannon	0.009	·	29.218	*P*<0.001	1.196	·
**Autumn**	***Fungi***	Richness	11.576	*P*<0.01	0.086	·	0.707	·
		Shannon	6.499	*P*<0.05	0.903	·	0.625	·
	Bacteria	Richness	2.942	·	0.288	·	0.476	·
		Shannon	3.062	·	0.008	·	1.315	·

Table S5. Three-way ANOVA of the alpha diversity (Richness and Shannon indexes) for fungal and bacterial communities in soils.

		**Fungi**		**Bacteria**	
		**Richness**	**Shannon**	**Richness**	**Shannon**
**Orchard (O)**	**F**	15.380	14.151	5.902	2.614
	**Sig.**	*P*<0.001	*P*<0.001	*P*<0.05	·
**Status (S)**	**F**	0.001	0.067	4.678	5.913
	**Sig.**	·	·	*P*<0.05	*P*<0.05
**Season (t)**	**F**	6.138	6.416	1.385	3.880
	**Sig.**	*P*<0.05	*P*<0.05	·	·
**Orchard×Status**	**F**	1.797	1.233	0.317	0.310
	**Sig.**	·	·	·	·
**Orchard×Season**	**F**	0.097	0.447	0.233	2.357
	**Sig.**	·	·	·	·
**Status×Season**	**F**	0.162	1.994	9.445	5.147
	**Sig.**	·	·	*P*<0.01	*P*<0.05
**Orchard×Status×Season**	**F**	0.087	0.023	0.370	2.274
	**Sig.**	·	·	·	·

Table S6. One-way ANOVA for the relative abundance of fungal communities at the phylum level.

	**Spring**				**Autumn**			
	**Orchard 1**		**Orchard 2**		**Orchard 1**		**Orchard 2**	
	**F**	**Sig.**	**F**	**Sig.**	**F**	**Sig.**	**F**	**Sig.**
Ascomycota	10.234	*P* ≤ 0.05	0.211	·	10.005	*P* ≤ 0.05	0.264	·
Basidiomycota	9.090	*P* ≤ 0.05	0.824	·	1.047	·	5.794	·
Rozellomycota	1.167	·	0.314	·	3.301	·	0.176	·
Chytridiomycota	0.118	·	0.791	·	0.231	·	2.154	·
Mortierellomycota	0.777	·	2.214	·	0.481	·	0.130	·
Glomeromycota	3.097	·	0.000	·	1.406	·	0.429	·
Mucoromycota	6.241	*P* ≤ 0.05	0.256	·	1.000	·	6.311	*P* ≤ 0.05
Aphelidiomycota	0.821	·	3.694	·	0.342	·	0.020	·
Kickxellomycota	0.054	·	0.048	·	0.047	·	0.591	·
Olpidiomycota	0.982	·	0.541	·	1.923	·	6.593	*P* ≤ 0.05
**Other**	0.626	·	0.002	·	0.038	·	0.436	·
**Unassigned**	6.676	*P* ≤ 0.05	0.281	·	4.225	·	0.437	·

Table S7. One-way ANOVA for the relative abundance of bacterial communities at the phylum level.

	**Spring**				**Autumn**			
	**Orchard 1**		**Orchard 2**		**Orchard 1**		**Orchard 2**	
	**F**	**Sig.**	**F**	**Sig.**	**F**	**Sig.**	**F**	**Sig.**
Proteobacteria	86.323	*P* ≤ 0.001	0.527	·	1.193	·	0.024	·
Planctomycetes	9.720	*P* ≤ 0.05	0.686	·	0.001	*P* ≤ 0.001	0.028	·
Actinobacteria	16.661	*P* ≤ 0.01	1.808	·	0.001	*P* ≤ 0.001	13.897	*P* ≤ 0.01
Firmicutes	22.222	*P* ≤ 0.01	4.506	·	0.015	*P* ≤ 0.05	8.694	*P* ≤ 0.05
Acidobacteria	48.948	*P* ≤ 0.001	0.587	·	0.277	·	3.036	·
Bacteroidetes	29.009	*P* ≤ 0.01	3.687	·	2.013	·	3.045	·
Verrucomicrobia	23.942	*P* ≤ 0.01	0.004	·	48.112	·	6.486	*P* ≤ 0.05
Chloroflexi	0.079	·	0.319	·	1.005	·	0.710	·
Armatimonadetes	34.041	*P* ≤ 0.01	0.001	·	6.141	·	0.753	·
Gemmatimonadetes	0.255	·	0.661	·	2.428	·	0.538	·

candidate_division_WPS-1	0.547	·	0.547	·	0.270	·	0.194	·
Thaumarchaeota	0.584	·	2.792	·	0.714	·	0.174	·
Nitrospirae	0.869	·	0.006	·	0.783	·	3.858	·
**Other**	4.162	·	2.246	·	14.862	·	0.585	·
**Unassigned**	2.743	·	8.793	$P \leq 0.05$	15.057	·	2.589	·
